# Supplementary material for: Two-dimensional ferroelectric channel transistors integrating ultra-fast memory and neural computing
Source: Nat Commun. 2021 Jan 4;12:53. doi: 10.1038/s41467-020-20257-2 (PMC7782550; doi:10.1038/s41467-020-20257-2)
Supplement: Supplementary file 1 — Supplementary Information [file 41467_2020_20257_MOESM1_ESM.pdf]

Supplementary Information

Two-dimensional ferroelectric channel transistors integrating ultra-fast memory and neural computing

Shuiyuan Wang[1], Lan Liu[1], Lurong Gan[1], Huawei Chen[1], Xiang Hou[1], Yi Ding[1], Shunli Ma[1], David Wei Zhang[1], Peng Zhou[1,*]

[1]ASIC & System State Key Lab., School of Microelectronics, Fudan University, Shanghai 200433, China

* Correspondence should be addressed to Peng Zhou: pengzhou@fudan.edu.cn

Supplementary Note 1: The schematic of fabricated 2D α-In_2Se_3 FeCTs process flow

Figure S1 shows the detailed preparation process of 2D α-In_2Se_3 FeCTs. First, Al_2O_3 is deposited on the substrate by ALD as the bottom dielectric layer, the bottom h-BN and 2D α-In_2Se_3 channel layer is prepared by mechanical exfoliation. Then PVA assists the transfer of h-BN as the top dielectric layer, wet-removes the PVA sacrificial layer, and then uses electron beam lithography to form the electrode pattern. It is worth mentioning that Al_2O_3 grown by ALD produces electrostatic doping to the α-In_2Se_3 channel,[1] so the bottom h-BN is needed to optimize the

interface. And the transferred top h-BN not only serves as a dielectric, but also provides passivation to the α-In_2Se_3 channel to isolate the influence of the ambient atmosphere.[4-6] Finally, source-drain and top gates are deposited by electron beam evaporation, and a heavily doped silicon substrate is used as the global gate.

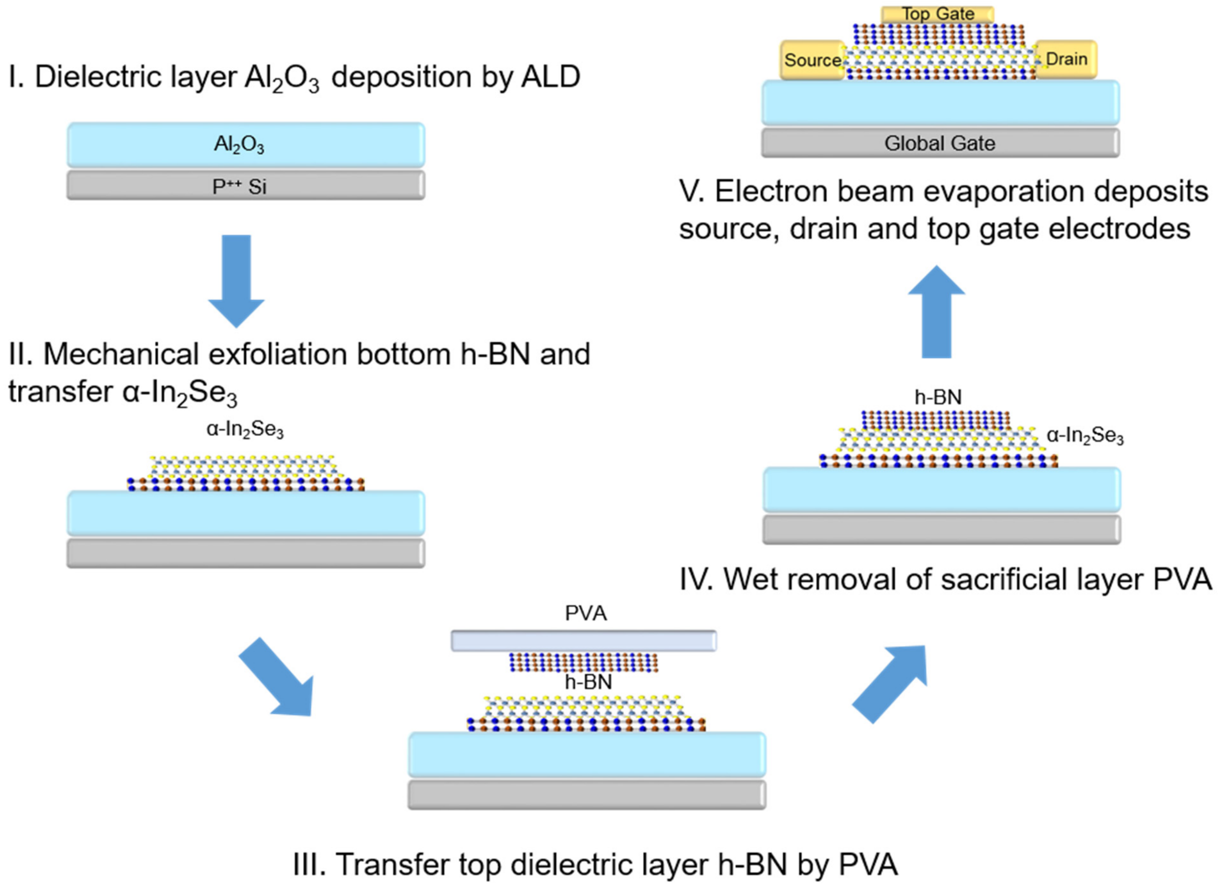

Figure S1. Schematic of fabricated process flow, including dielectric layer deposition, bottom h-BN mechanical exfoliation, α-In_2Se_3 channel layer and top h-BN dielectric layer transfer, transfer sacrificial layer removal and electrodes deposition.

Supplementary Note 2: XRD characterization of α-In_2Se_3

Figure S2 shows the XRD characterization of α-In_2Se_3 crystals used for 2D FeCTs, where the diffraction pattern only shows a *c*-plane peak and its high-ordered interplanar spacing. The peak pattern enables the determination of the lattice constant *c* (≈19.23 Å), which is highly consistent with the reported 2H α-In_2Se_3 and is significantly different from 3R α-In_2Se_3.[2,3]

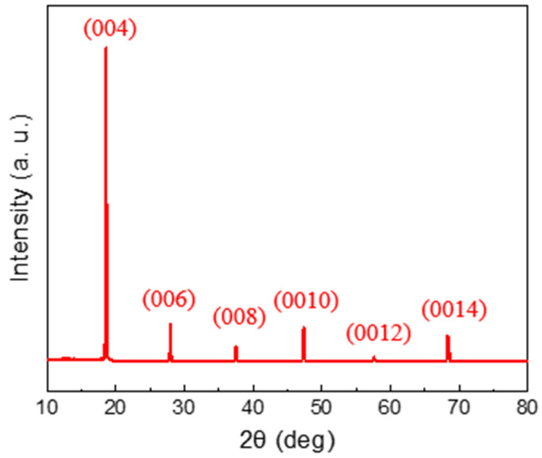

Figure S2. XRD characterization of the α-In_2Se_3 crystal, where the diffraction pattern only shows the *c*-plane peak and its higher-order interplanar spacing.

Supplementary Note 3: 2D FeCTs morphology and Raman characterization

Figure S3a characterizes the surface morphology of 2D FeCTs by AFM, where the height of the channel α-In_2Se_3, the top and bottom h-BN are about 40 nm, 20 nm, 20 nm, respectively. **Figure S3b** shows the Raman spectrum of h-BN with a clear 1366 cm^{-1} peak position, which is consistent with previous reports.[4]

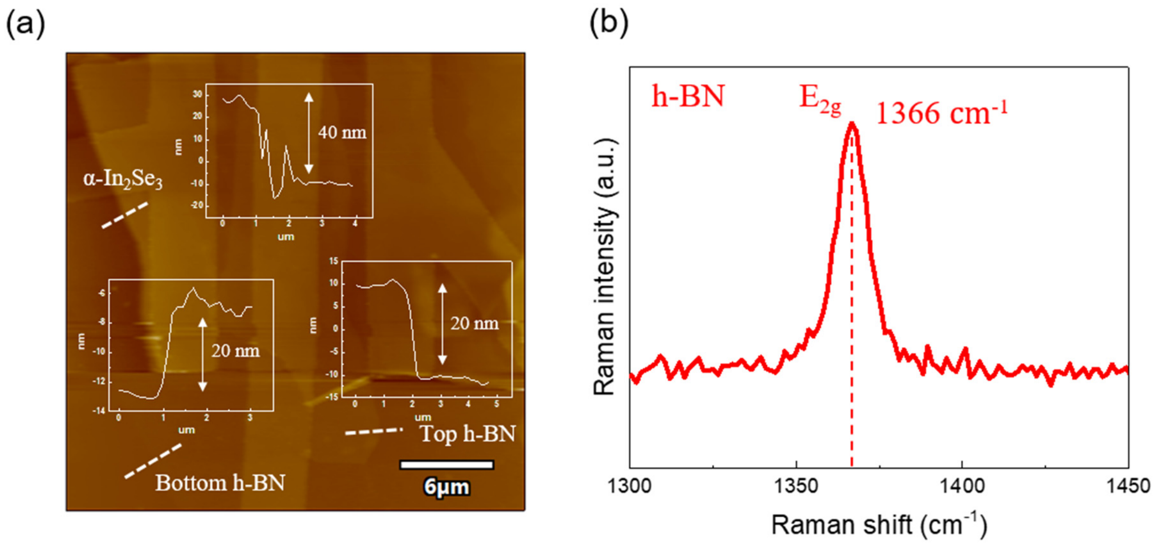

Figure S3. 2D FeCTs surface morphology and Raman characterization. (a) AFM height error graph of 2D FeCT surface topography, indicating that the thickness of α-In_2Se_3, top and bottom h-BN is 40 nm, 20 nm, 20 nm. (b) Raman characterization of h-BN, which shows a clear peak position at 1366 cm^{-1}.

Supplementary Note 4: On-field PFM amplitude and phase hysteresis loops of the channel α-In_2Se_3

Figure S4a depicts the on-field PFM amplitude versus voltage bias hysteresis loop of the channel α-In_2Se_3 with three cycles. **Figure S4b** shows the on-field PFM phase versus voltage bias hysteresis loop of the channel α-In_2Se_3 with three cycles. Both the PFM amplitude and phase hysteresis loops show that the channel α-In_2Se_3 exhibits a clear ferroelectric polarization reversal effect under the external electric field.

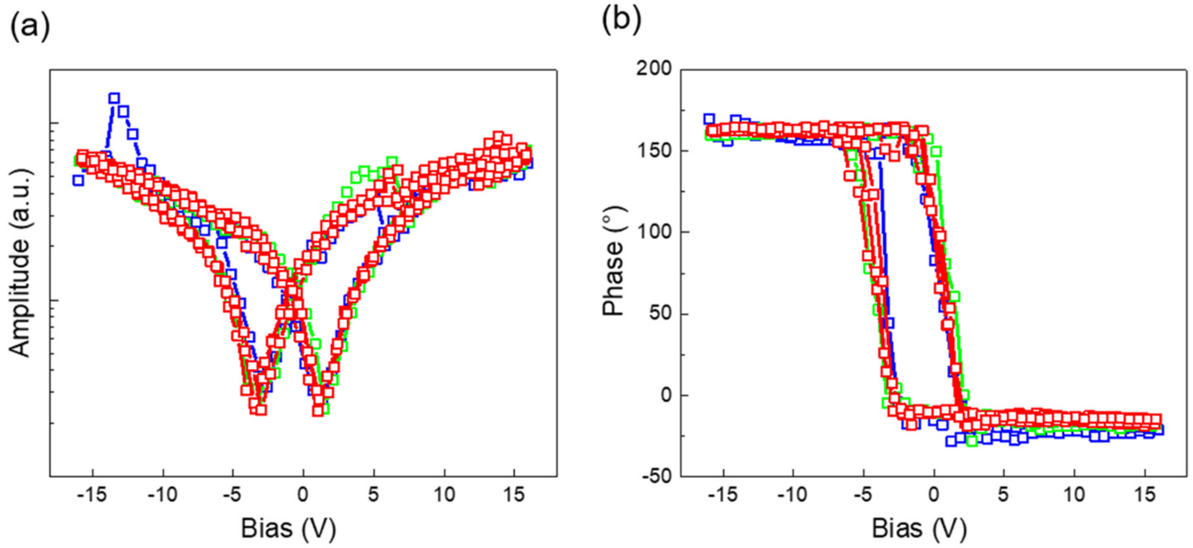

Figure S4. (a) On-field PFM amplitude versus voltage bias hysteresis loop with three cycles. (b) On-field PFM phase versus voltage bias hysteresis loop with three cycles.

Supplementary Note 5: α-In_2Se_3 ferroelectric hysteresis curve with varying scanning voltage and 2D FeCTs dynamic equilibrium energy band diagram

Figure S5 shows the energy band diagram of FeCTs in equilibrium, where 2D α-In_2Se_3, as the essence of a ferroelectric semiconductor, has mobile charges in addition to the polarization bound charges.[1,7] Besides, band bending and polarization states are simultaneously modulated by top and global gates.

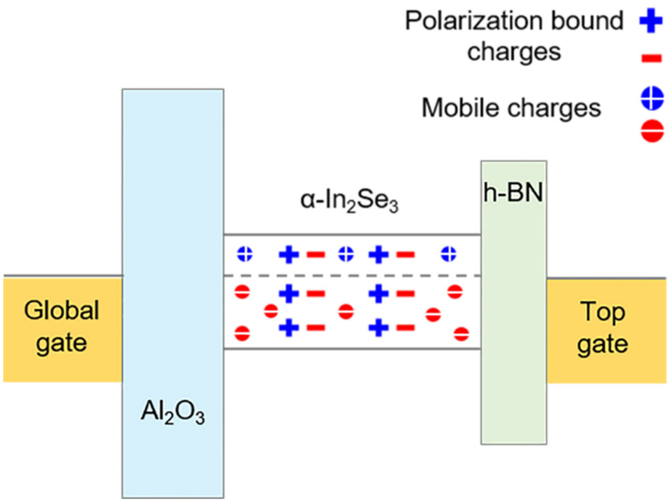

Figure S5. Equilibrium band diagram of 2D α-In_2Se_3 FeCTs. It is worth noting that 2D α-In_2Se_3, which is a ferroelectric semiconductor, has both mobile charges and polarization bound charges. And band bending and ferroelectric polarization are modulated by top and global gates.

Supplementary Note 6: Output curves under varying VGG and VTG

Figure S6 shows the output curves under global and top gate. The output current under both GG and TG modulation increases with the accumulated gate bias. However, the drain current of GG modulation is relatively larger than that of TG, which may be due to the lower coverage of the positive electric field induced by TG (because of the top dielectric layer hBN may break down under a large forward electric field) and the weaker tunability of TG than GG.

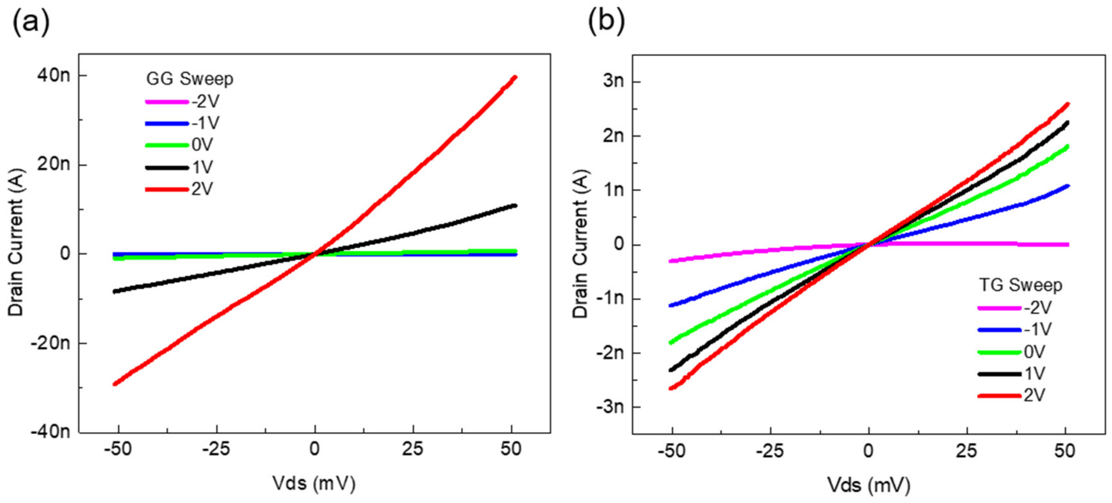

Figure S6. Output curves under varying VGG and VTG, Vds sweep is fixed from -50 to 50 mV. (a) Output curves under GG modulation, where GG is from -2 to 2 V in 1 V steps. (b) Output curves under TG modulation, where TG is from -2 to 2 V in 1 V steps.

Supplementary Note 7: Memory LRS/HRS and dynamic erase/write characteristics

Figure S7 depicts the LRS and HRS under NVM basic erase and write operations, and the corresponding dynamic characteristics. **Figure S7a** shows the 2D α-In_2Se_3 FeCTs based NVM LRS and HRS implemented in sequence under the -6 V GG erase and +6 V GG write operations, with Vds of 0.1 V and VTG of 0 V, showing non-volatile data retention. **Figure S7b** shows the robust 10-cycle dynamic erase/write current response of FeCTs NVM, where the spike voltage is ±8 V, Vds is 0.1 V and VTG is 0 V.

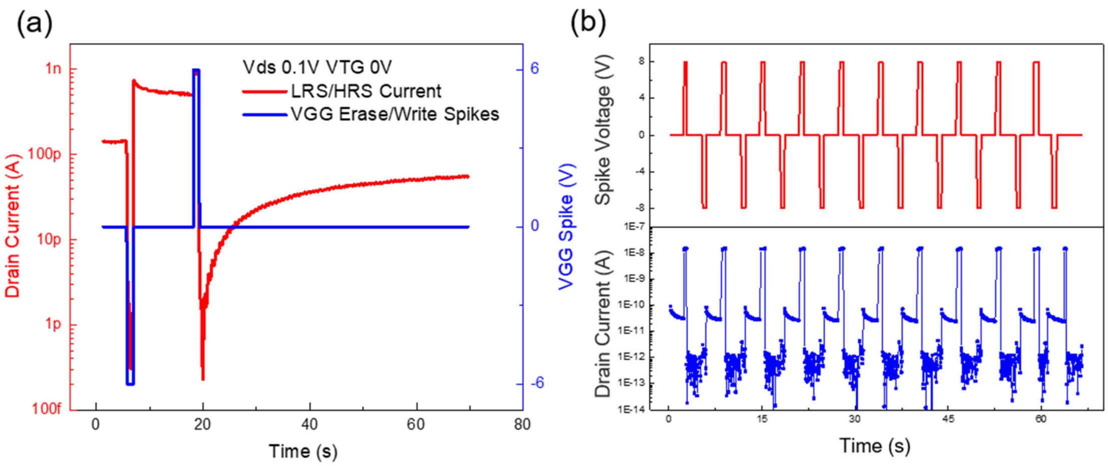

Figure S7. Memory LRS/HRS and dynamic erase/write characteristics. (a) The NVM LRS and HRS realized by VGG erase and write spikes, Vds is fixed at 0.1 V and VTG is 0 V. (b) The dynamic erase/write response of FeCTs NVM.

Supplementary Note 8: Top gate transfer and minimal spike LTP/LTD curves

Figure S8a shows a typical TG transfer curve with Vds of 1 V and VGG of 0 V, where the red curve is the drain current and the black curve is the TG leakage current. The TG transfer curve of 2D α-In_2Se_3 FeCTs also shows a significant clockwise hysteresis memory window, similar to GG, and compared to the channel current, the leakage current is negligible, reflecting the excellent dielectric properties of TG hBN. **Figure S8b** exhibits the original voltage-current curve of progressive excitatory and inhibitory PSC modulation achieved with minimal spike voltage of ±0.5 V. The red curve is PSC under LTP and LTD, sequentially, and the black curve is the corresponding VG spike. It is worth noting that the excitation and inhibition modulation with minimum spike voltage indicates the extremely low power consumption of 2D α-In_2Se_3 FeCTs for neural computing.[8]

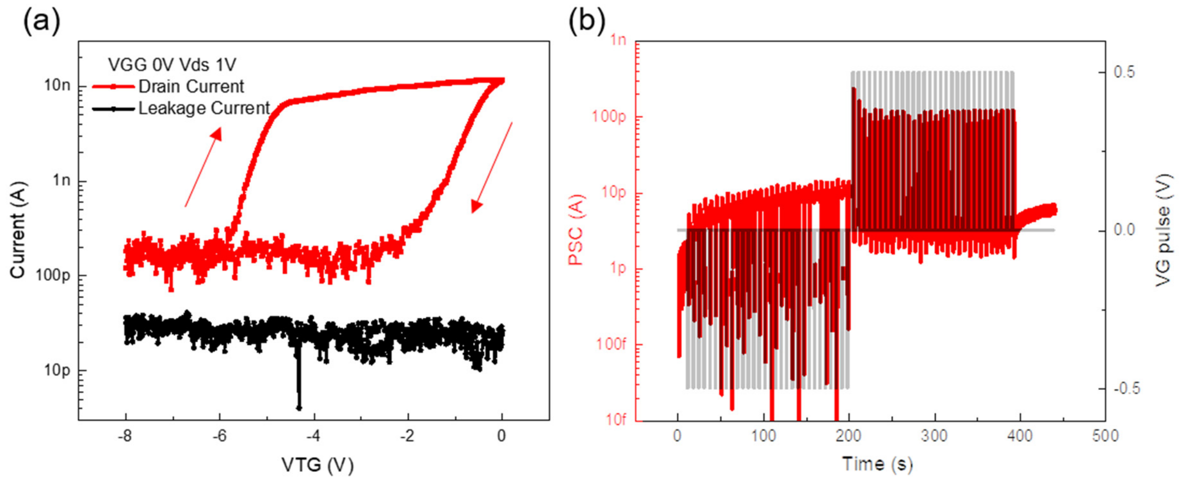

Figure S8. Top gate transfer and minimal spike LTP/LTD curves. (a) TG transfer curve with Vds of 1 V and VGG of 0 V, which shows a significant clockwise hysteresis window and negligible leakage current. (b) Progressive excitatory and inhibitory PSC modulation achieved with minimal spike voltage of ±0.5 V, which corresponds to LTP and LTD, respectively. The red curve is the modulated PSC under LTP and LTD, and the black curve is the corresponding VG spike.

Supplementary Note 9: Inhibitory PSC induced paired pulse facilitation characteristics

Figure S9 describes the characteristics of inhibitory PSC induced PPF in neural computing. As the spike interval increases, inhibitory PSC induced PPF index gradually returns to 100%, showing the disappearance of the short-term inhibitory effect. The PSC response after paired pulses is shown in **Figure S9a**. And the PSC amplitude after first spike is recorded as A_1, and the amplitude after second spike is recorded as A_2, and when A_2 is lower than A_1, the synaptic inhibition strengthening effect is simulated.[7] **Figure S9b** shows the inhibitory PSC induced PPF index as a function of interval time, which gradually refresh to 100% as the interval accumulates, corresponding to a typical STP in neural computing.[4,9]

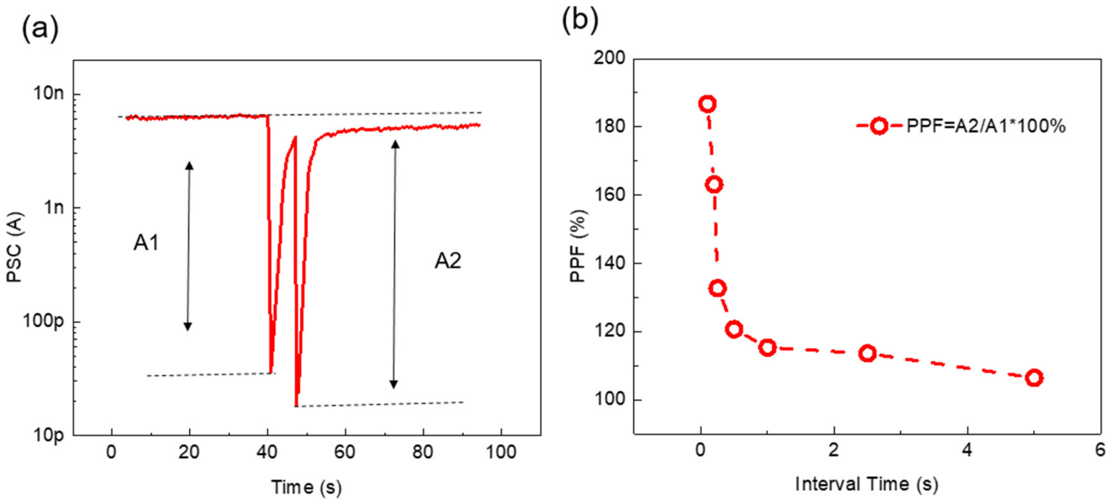

Figure S9. Paired pulse facilitation characteristics. (a) Typical PSC response to paired pulses. (b) PPF index as a function of interval time, which gradually refresh to 100% as the interval accumulates. And PPF index is calculated by A_2/A_1.

Supplementary Note 10: Thermal dependence of VGG output and transfer curves

Figure S10a shows the output characteristics (Vds from -1 to 1 V, VGG and VTG are fixed at 0 V) as a function of thermal temperature (298~423 K). As the temperature increases, the current climbs and a better contact is formed (The calculated barrier height is about 450.9 meV), which may be attributed to thermally affected channel ferroelectric polarization[10] and defect healing.[11,12] The thermal temperature-dependent transfer curves (VGG=-8~8 V, Vds=1 V) are shown in **Figure S10b**. Similarly, it still has clear memory windows, and the on-state current significantly increases to approximately the same value, while the off-state current gradually increases with increasing temperature, which shows flexible thermal temperature tunability.

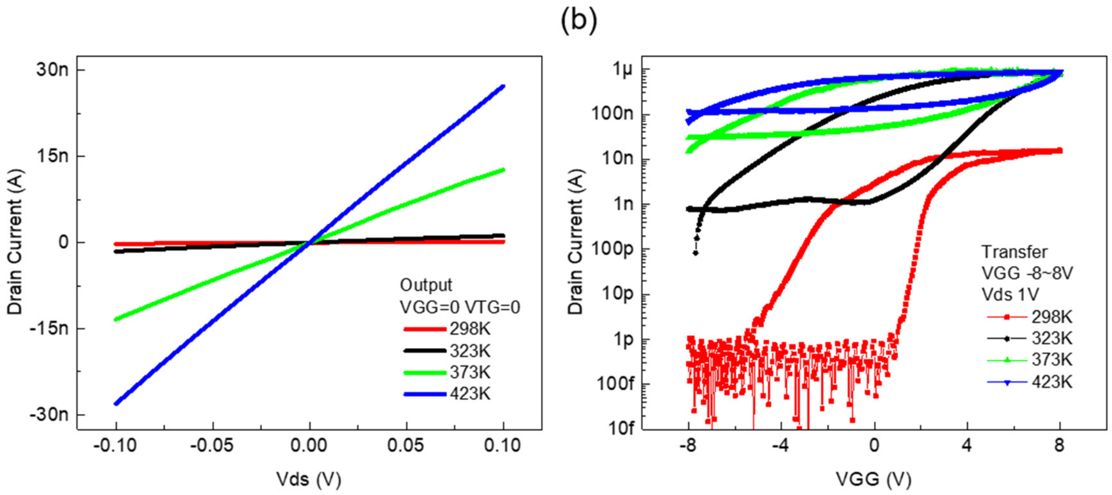

Figure S10. Thermal dependence of VGG output and transfer curves. (a) Thermal temperature-dependent output characteristics, where Vds is from -0.1 to 0.1 V, VGG and VTG are fixed at 0 V. As the temperature increases, the current increases and a more symmetrical ohmic contact is formed, which is consistent with the optimization of contact and channel defects after annealing. (b) The transfer curves as a function of thermal temperature, with VGG from -8 to 8 V and Vds of 1 V. It still shows clear memory windows, and the on-state current increases significantly to an approximate value of 1 μA, while the off-state current gradually climbs as the temperature increases.

Supplementary Note 11: Thermal dependence of VGG transfer curves with varying sweeping range

Figures S11a, b depicts the thermal dependence of the transfer curves in the VGG range of -4 to 4 V and -6 to 6 V, respectively. Similar to **Figure S10b**, as the temperature increases from 298 to 373 K, the on-state current increased significantly to an approximate value, while the off-state

current gradually increased, and 2D α-In_2Se_3 FeCTs shows a varying memory window, reflecting flexible thermal temperature adjustability.

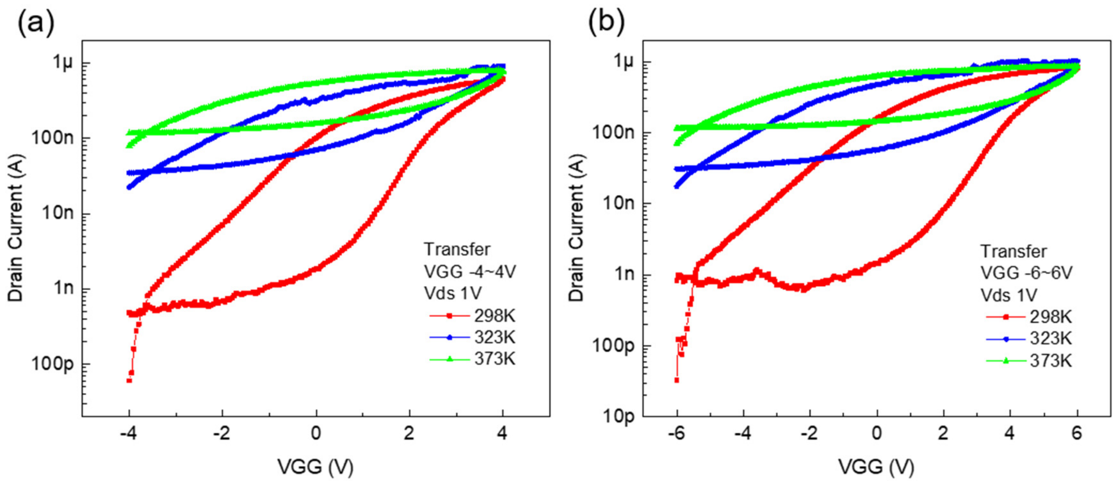

Figure S11. Thermal dependence of VGG transfer curves with varying sweeping range. (a) Thermal dependence of the transfer curve in the VGG range of -4 to 4 V, where Vds is fixed at 1 V and the thermal temperature is from 298 to 373 K. (b) Vds and thermal temperature fixed, thermal-dependent transfer curve under -6 ~ 6 V VGG sweep.

Supplementary Note 12: Different VGG transfer curves under 323 K and memory LRS/HRS thermal tunability

Figure S12a shows the transfer curves of VGG sweep range of -4 ~ 4 V, -6 ~ 6 V and -8 ~ 8 V at 323 K. As the VGG voltage increases, the hysteresis memory window expands, and the ON/OFF ratio can still reach ~10^3 at 323 K. **Figure S12b** depicts the thermal temperature-adjustable LRS and HRS of 2D FeCTs NVM under ±4 V erase and write spikes. As the thermal temperature increases, both LRS and HRS increase and gradually overlap, as shown by the red and blue arrows

in **Figure S12b**, which is consistent with the raise of the switching coefficient under thermal operation, indicating the depolarization of α-In_2Se_3 channel ferroelectric and the electrothermal tunability of FeCTs NVM.[13]

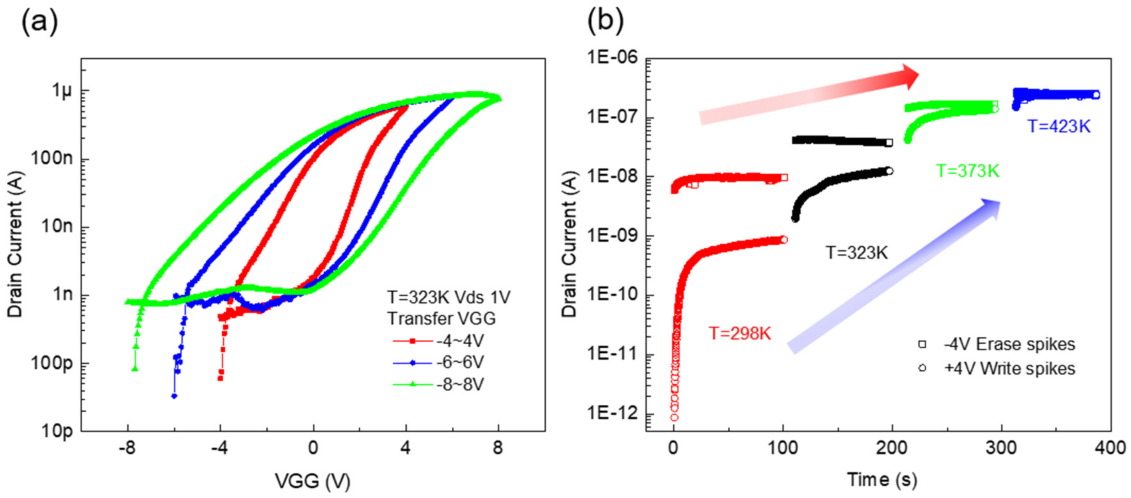

Figure S12. Different VGG transfer curves under 323 K and memory LRS/HRS thermal tunability. (a) The transfer curves of VGG sweep range of -4 ~ 4 V, -6 ~ 6 V and -8 ~ 8 V at 323 K, and Vds is fixed at 1 V. (b) 2D FeCTs NVM thermally tunable LRS and HRS under ±4 V erase and write spikes, Vds is fixed at 1 V. The evolution trend of LRS and HRS indicated by red and blue arrows implies ferroelectric channel depolarization and electrothermal adjustability.

Supplementary Note 13: Flow chart of simulated Iris recognition and classification based on FeCTs fully connected neural network

Figure S13 shows the detailed flow chart of iris recognition and classification based on FeCTs simulated neural network, including loading the iris standard dataset, defining a fully connected network model, training the network, testing the network, and obtaining the weight matrix under

the optimal accuracy. Subsequently, the device conductance mapping function SME is defined, the conductance is mapped to the optimal weight matrix, the new network after the conductance mapping is obtained, and the recognition and classification accuracy of the new network is retested.

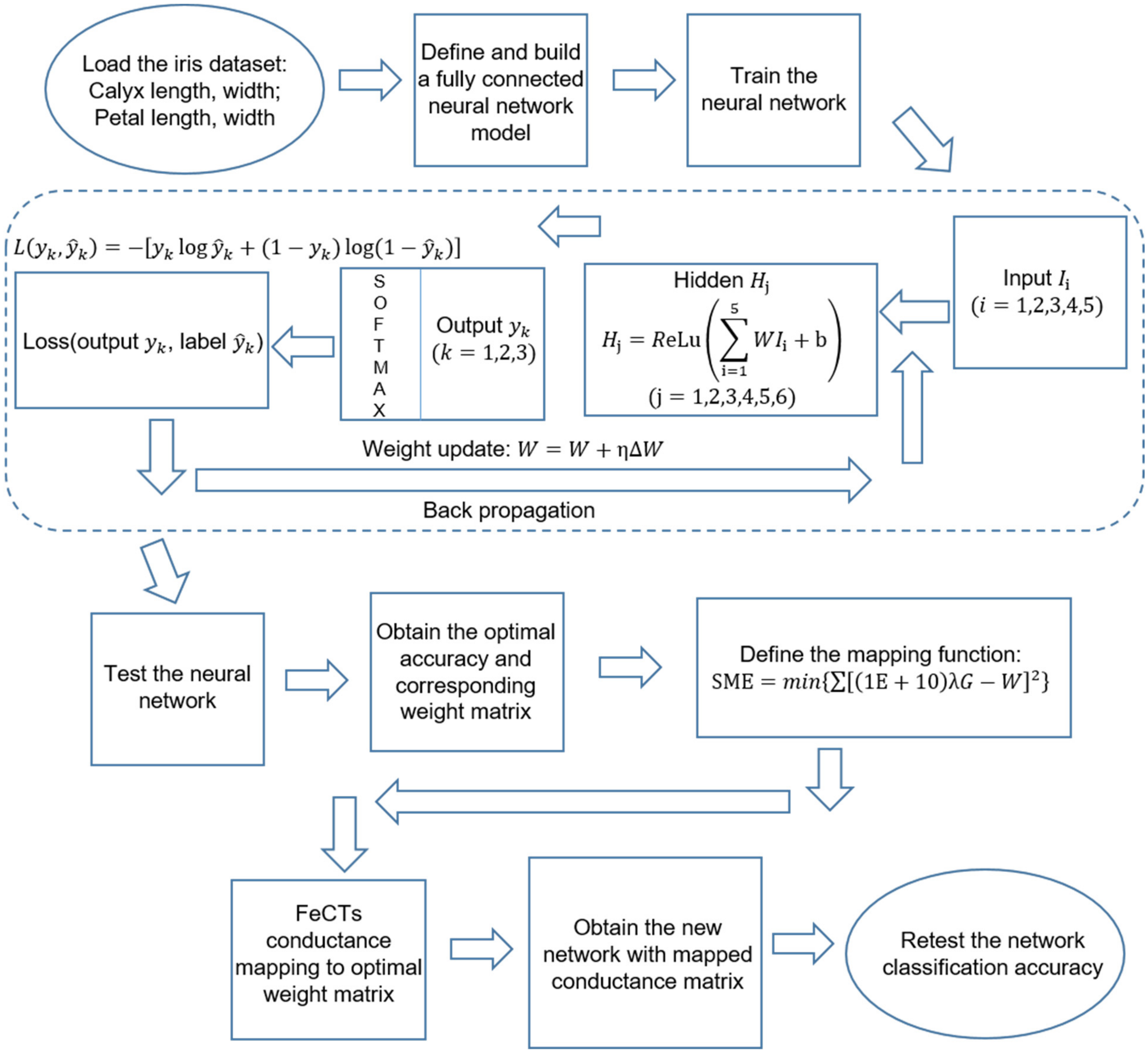

Figure S13. Flow chart of iris classification based on FeCTs simulated neural network.

Supplementary Note 14: SME with varying thermal and spike amplitudes to determine mapping coefficient

Figure S14a depicts the SME as a function of mapping coefficient at varying thermal temperatures. The optimal mapping coefficients λ at 323 K, 373 K, and 423 K are 0.18, 0.28, and 0.9, respectively. **Figure S14b** shows the SME as a function of mapping coefficient with varying spike amplitudes at RT. And the optimal mapping coefficients λ for ±4 V and ±0.5 V spikes at RT are 8 and 12.5, respectively. Both thermal temperature and spike amplitudes have an effect on the mapping coefficient, due to the modulation of α-In_2Se_3 FeCTs channel polarization, which in turn affects SME.

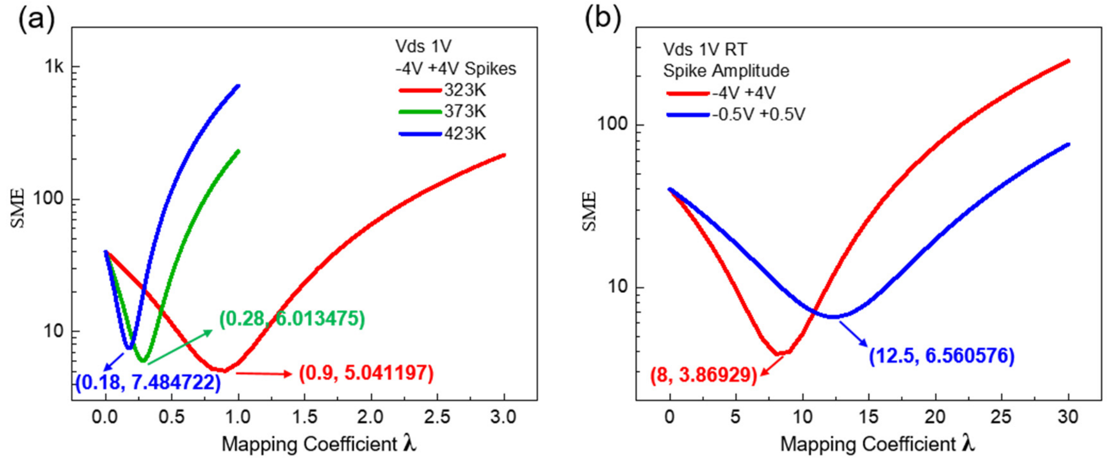

Figure S14. SME with varying thermal and spike amplitudes to determine mapping coefficient. (a) SME as a function of mapping coefficient at varying thermal temperatures (323 K, 373 K, 423 K). (b) SME as a function of mapping coefficient with varying spike amplitudes (±4 V, ±0.5 V) at RT.

Supplementary Note 15: Weight distribution of conductance mapping with varying spike amplitude at RT

Figure S15 shows the weight distribution of conductance mapping with varying spike amplitudes at RT. Specifically, **Figure S15a** shows the network weight distribution after the α-In_2Se_3 FeCTs conductance mapping of ±4 V spikes at RT, while **Figure S15b** exhibits the ±0.5 V spikes mapped weight distribution.

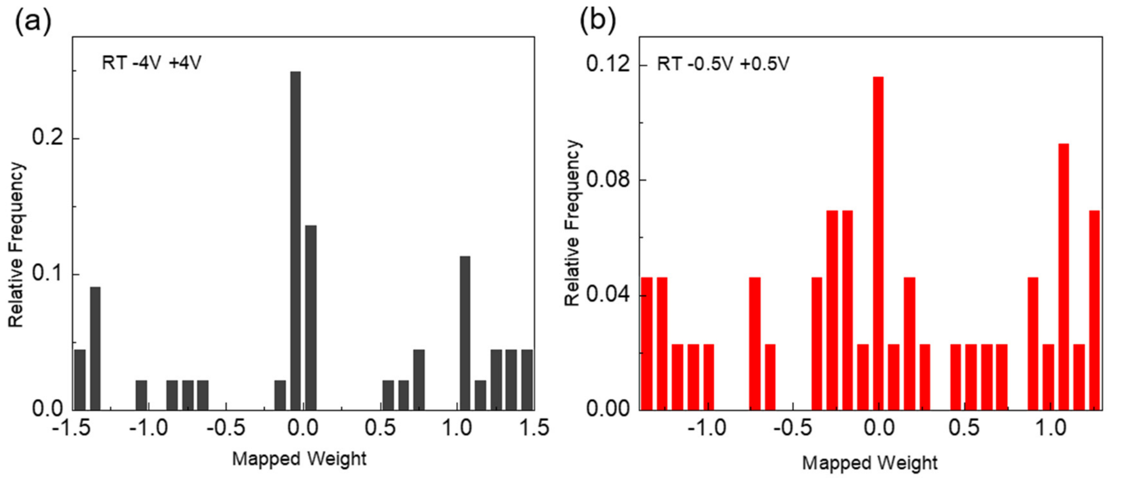

Figure S15. Weight distribution of conductance mapping with varying spike amplitude at RT. (a) The network weight distribution after the FeCTs conductance mapping of ±4 V spikes. (b) The weight distribution of FeCTs conductance mapping under ±0.5 V spikes.

Supplementary Note 16: ALD 30 nm Al_2O_3 I-V and C-V characteristics

Figure S16a shows the I-V characteristics for negative and positive voltage sweeping, showing low leakage currents in the Al_2O_3 dielectric layer. Figure S16b shows the C-V characteristics for

different voltage sweep ranges, and the inset illustrates the constructed parallel-plate capacitor structure. And we have calculated the dielectric constant of the deposited Al_2O_3 to be about 9.

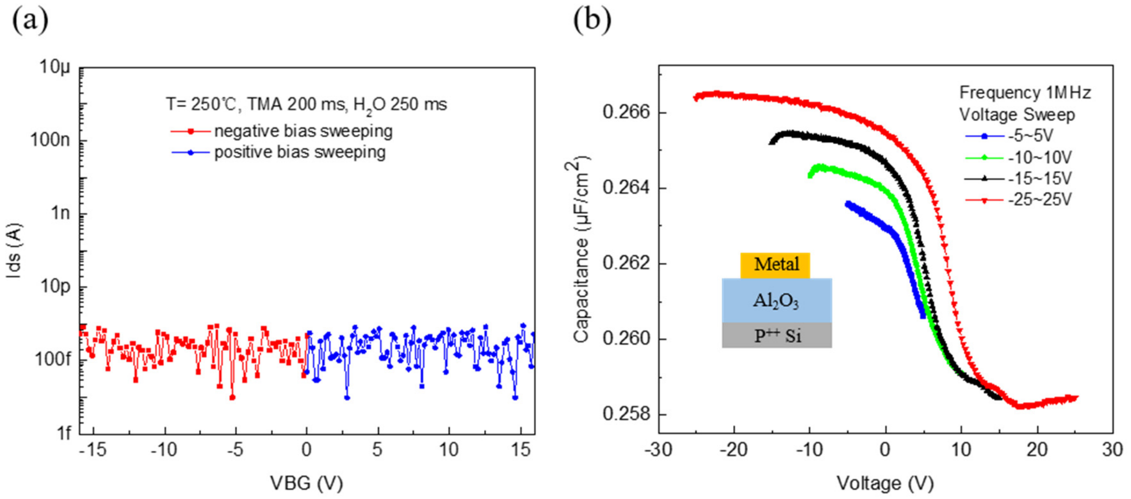

Figure S16. ALD 30 nm Al_2O_3 I-V and C-V characteristics. (a) The I-V characteristics under positive and negative bias sweeping. (b) The C-V characteristics for different voltage sweep ranges, and the inset illustrates the constructed parallel-plate capacitor structure.

References:

1. Si, M. *et al.* A ferroelectric semiconductor field-effect transistor. *Nature Electronics* **2**, 580-586 (2019).
2. Ho C. *et al.* Surface oxide effect on optical sensing and photoelectric conversion of α-In2Se3 hexagonal microplates[J]. *ACS applied materials & interfaces*, **5**, 2269-2277 (2013).
3. Jacobs-Gedrim R. *et al.* Extraordinary photoresponse in two-dimensional In_2Se_3 nanosheets[J]. *ACS nano*, **8**, 514-521 (2014).
4. Wang, S. *et al.* A Photoelectric-Stimulated MoS2 Transistor for Neuromorphic Engineering. *Research* **2019**, 1618798 (2019).

5. Lee, G.-H. *et al.* Highly stable, dual-gated MoS2 transistors encapsulated by hexagonal boron nitride with gate-controllable contact, resistance, and threshold voltage. *ACS Nano* **9**, 7019-7026 (2015).
6. Petrone, N. *et al.* Flexible graphene field-effect transistors encapsulated in hexagonal boron nitride. *ACS Nano* **9**, 8953-8959 (2015).
7. Cui, C. *et al.* Intercorrelated in-plane and out-of-plane ferroelectricity in ultrathin two-dimensional layered semiconductor In2Se3. *Nano Lett.* **18**, 1253-1258 (2018).
8. Van De Burgt, Y., Melianas, A., Keene, S. T., Malliaras, G. & Salleo, A. Organic electronics for neuromorphic computing. *Nature Electronics* **1**, 386-397 (2018).
9. Wang, S. *et al.* A MoS2/PTCDA hybrid heterojunction synapse with efficient photoelectric dual modulation and versatility. *Adv. Mater.* **31**, 1806227 (2019).
10. Xue, F. *et al.* Gate-Tunable and Multidirection-Switchable Memristive Phenomena in a Van Der Waals Ferroelectric. *Adv. Mater.* **31**, 1901300 (2019).
11. Jia, K. *et al.* Effects of defects and thermal treatment on the properties of graphene. *Vacuu* **116**, 90-95 (2015).
12. Xin, G. *et al.* Advanced phase change composite by thermally annealed defect-free graphene for thermal energy storage. *ACS applied materials & interfaces* **6**, 15262-15271 (2014).
13. Xue, F. *et al.* Gate-Tunable and Multidirection-Switchable Memristive Phenomena in a Van Der Waals Ferroelectric. *Adv. Mater.* **31**, 1901300 (2019).
